# Supplementary material for: Non-Oncogene Addiction of KRAS-Mutant Cancers to IL-1β via Versican and Mononuclear IKKβ
Source: Cancers (Basel). 2023 Mar 20;15(6):1866. doi: 10.3390/cancers15061866 (PMC10047096; doi:10.3390/cancers15061866)
Supplement: Supplementary file 1 [file cancers-15-01866-s001.zip › Spella et al_Cancers_Supplementary Materials.pdf]

Supplementary Materials for

Non-oncogene addiction of *KRAS*-mutant cancers to IL-1β via versican and mononuclear IKKβ

Magda Spella, Giannoula Ntaliarda, Georgios Skiadas, Anne-Sophie Lamort, Malamati Vreka, Antonia Marazioti, Ioannis Lilis, Eleni Bouloukou, Georgia A. Giotopoulou, Mario A.A. Pepe, Stefanie A.I. Weiss, Agnese Petrera, Stefanie M. Hauck, Ina Koch, Michael Lindner, Rudolph A. Hatz, Juergen Behr, Kristina A.M. Arendt, Ioanna Giopanou, David Brunn, Rajkumar Savai, Dieter E. Jenne, Maarten de Château, Fiona E. Yull, Timothy S. Blackwell, and Georgios T. Stathopoulos

Correspondence to: magsp@upatras.gr

This PDF file includes:

Figures S1 to S17
Tables S1 to S5
Caption for Data S1

Other Supplementary Materials for this manuscript include the following:

Data S1 [Excel file of proteomic analysis of secreted proteins of *Kras*-mutant and -wild-type cancer cells]

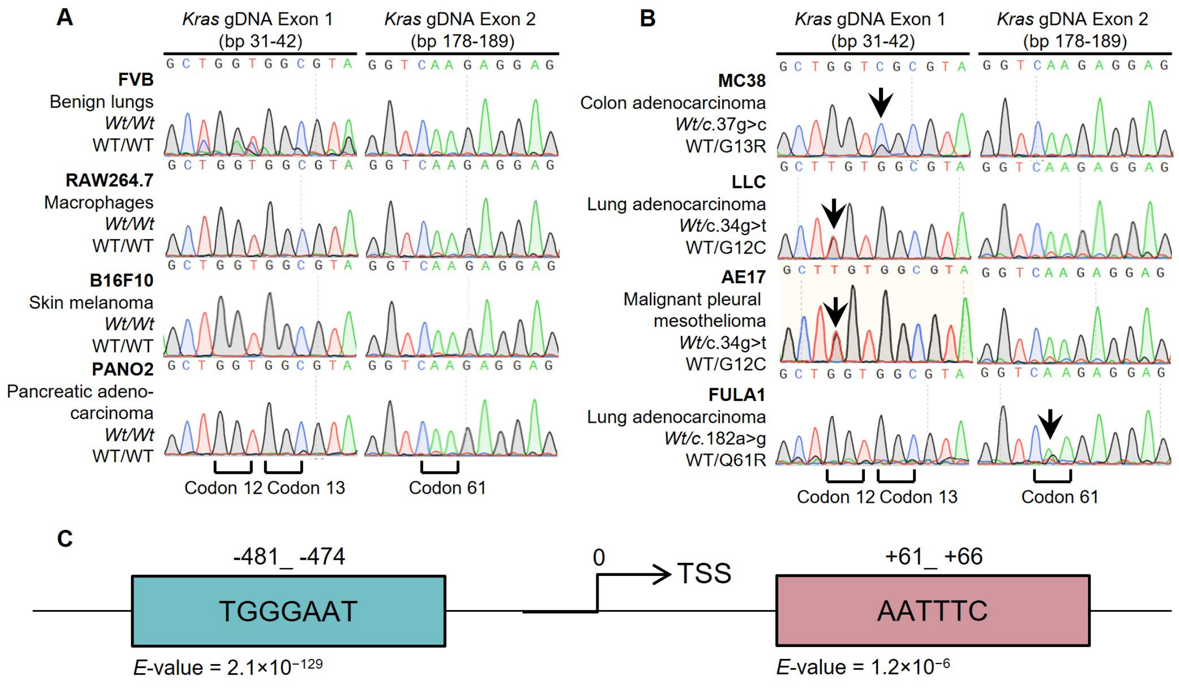

Figure S1. Seven murine cell lines with different *Kras* alleles and transcriptional control of interleukin (IL)-1β by nuclear factor (NF)-κB. (**A,B**) Sanger sequencing traces for *Kras* codons 12, 13, and 61 of all cell lines used. Arrows point to *Kras* mutations: MC38 cells carry G13R, LLC and AE17 cells G12C, FULA1 cells Q61R (B), and all other cell lines wild-type (*Wt*/WT; A) *Kras* alleles. Note that all mutant *Kras* alleles are in heterozygosity to *Wt* alleles. (**C**) *IL1B* as a NF-κB target gene in ChIPseq datasets from the CHEA Transcription Factor Targets dataset (https://maayanlab.cloud/Harmonizome/dataset/CHEA+Transcription+Factor+Targets). We downloaded the RELA (red) and RELB (blue) binding sequence motifs from the ENCODE portal (https://www.encodeproject.org/) with the identifiers: ENCFF507YCV (CHIP-seq on HuH-7.5 cells) and ENCFF615HZF (CHIP-seq on 8988T cells), respectively. E-value represents the statistical significance of the motif in terms of probability to be found in similarly sized set of random sequences. TSS: Transcription Start Site.

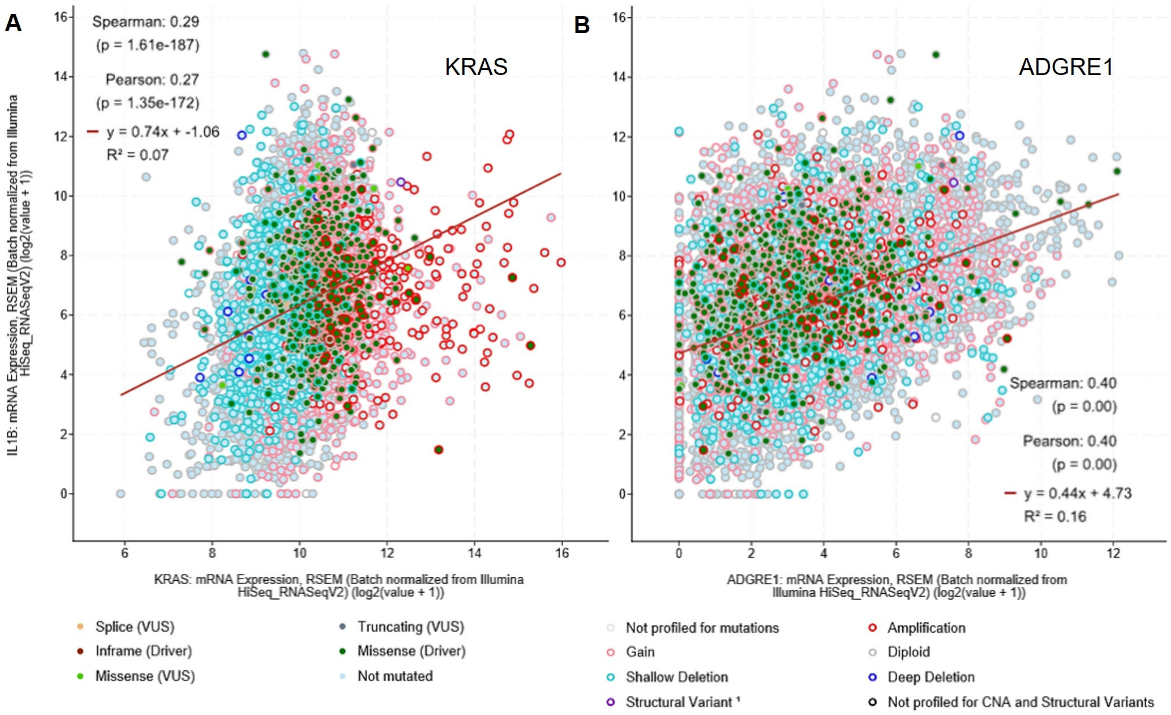

Figure S2. Expression of *IL1B* in correlation with *KRAS* and the macrophage marker *ADGRE1* in human tumors. Gene expression data from the cancer genome atlas (TCGA) pan-cancer dataset (n = 10,071 patients). Correlations of *IL1B* with *KRAS* (**A**) and the macrophage marker *ADGRE1* (encoding F4/80) (**B**) mRNA levels. Shown are raw data points (circles) color-coded by *KRAS* alteration status, regression lines and formulas (lines), as well as Spearman's and Pearson's correlation coefficients with probabilities (P) and squared correlation coefficients (R^2). Data from https://www.cbioportal.org/.

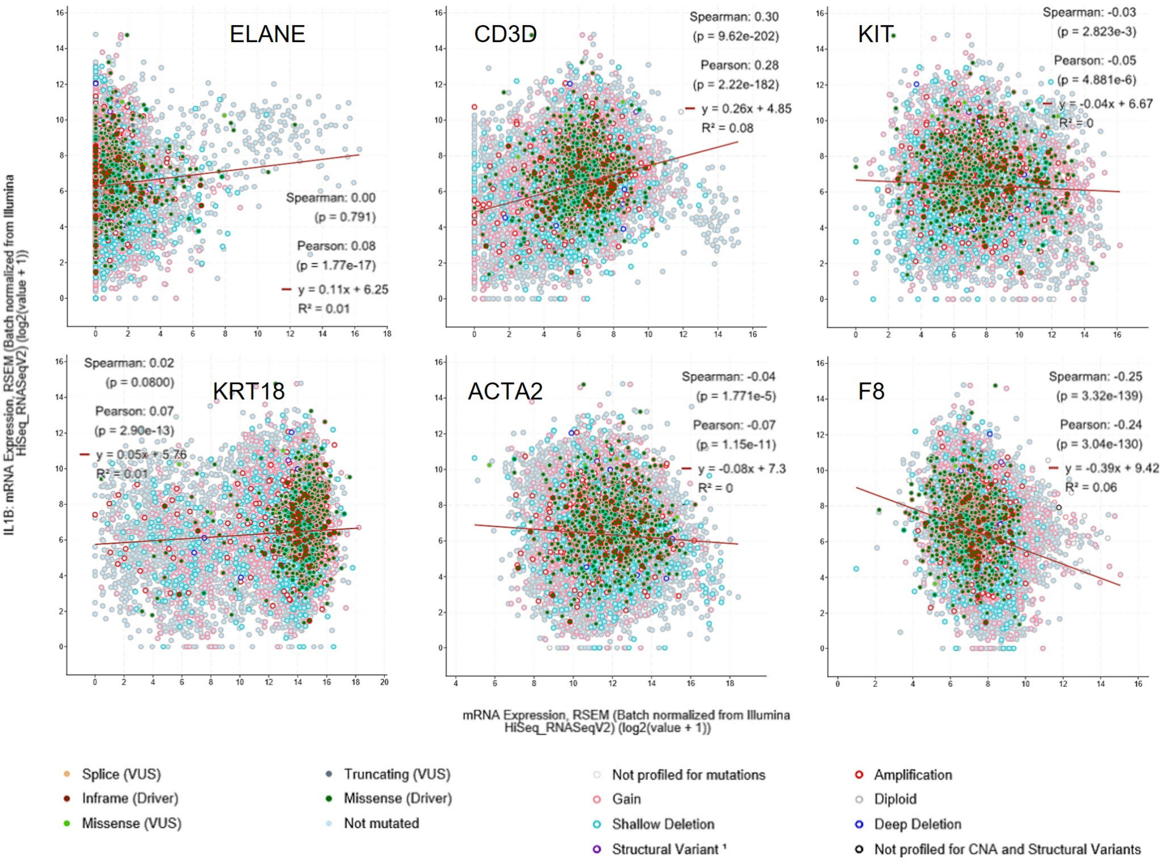

Figure S3. Expression of *IL1B* in correlation with lineage-specific markers in human tumors. Gene expression data from the cancer genome atlas (TCGA) pan-cancer dataset (*n* = 10,071 patients). Correlations between mRNA levels of *IL1B* and the neutrophil marker *ELANE* (top left; encoding neutrophil elastase), the pan-lymphocyte marker *CD3D* (top middle; encoding cluster of differentiation 3), the mast cell marker *KIT* (top right; encoding c-KIT), the cancer cell marker *KRT18* (bottom left; encoding cytokeratin 18), the fibroblast marker *ACTA2* (bottom middle; encoding α-smooth muscle actin), and the endothelial marker *F8* (bottom right; encoding factor VIII). Shown are raw data points (circles) color-coded by *KRAS* alteration status, regression lines and formulas (lines), as well as Spearman's and Pearson's correlation coefficients with probabilities (*P*) and squared correlation coefficients (R^2). Data from https://www.cbioportal.org/.

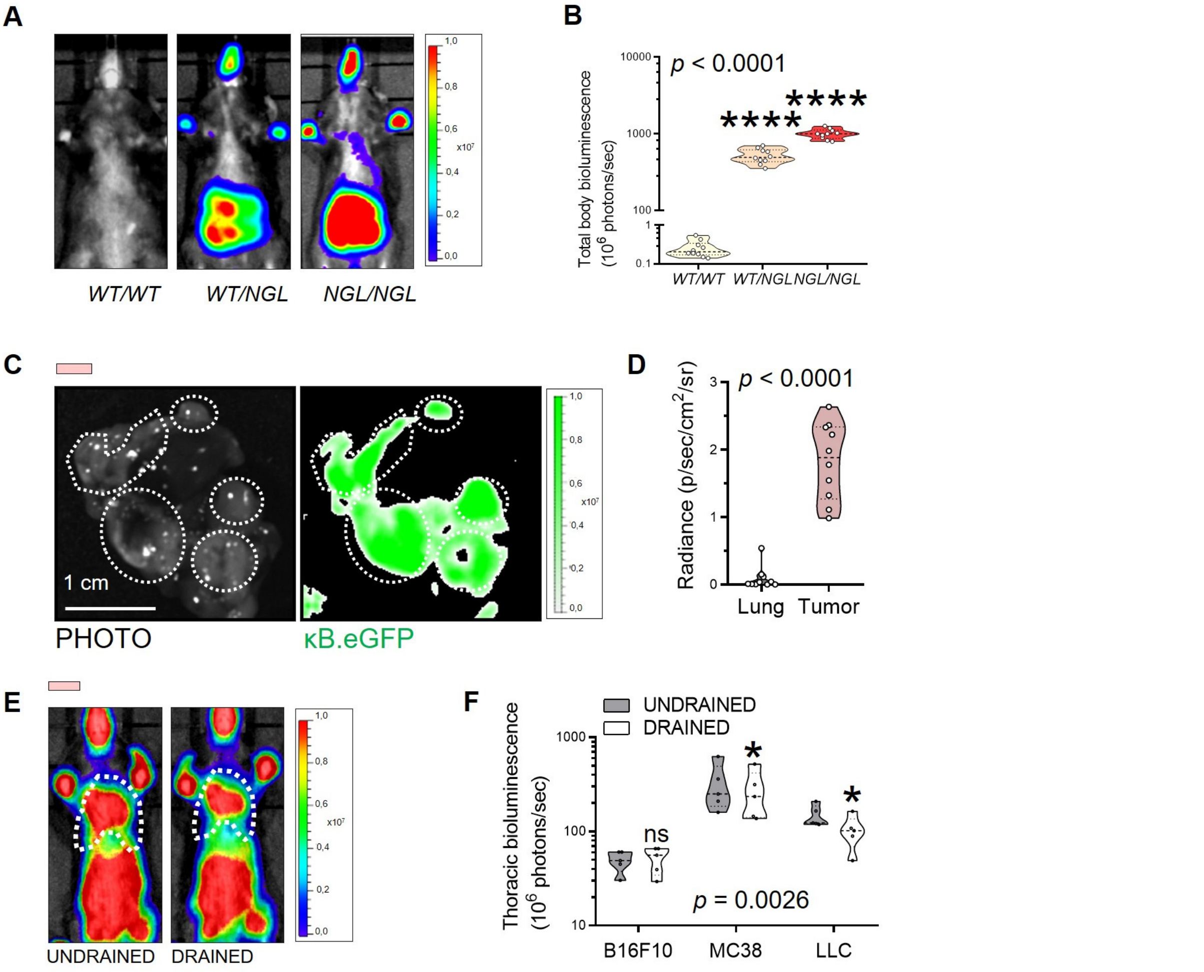

Figure S4. NF-κB activation in pleural metastases of *NGL* mice. (**A, B**) Representative photographic/bioluminescent images with pseudocolor scale (**A**) and data summary (**B**) from wild-type (*WT/WT*), heterozygote (*WT/NGL*), and homozygote (*NGL/NGL*) *NF-κB.eGFP.LUC* reporter mice at 5 min post-retroorbital injection of 1 mg D-Luciferin. Sample size (n) = 10/group; P, probability, one-way ANOVA; ****, $P < 0.0001$ compared with *WT/WT* mice, Bonferroni post-test. (**C, D**) Representative photographic/biofluorescent (**C**) images with pseudocolor scale and data summary (**D**) of κB.eGFP reporter signal (green) of lung explants of *NGL* mice at 14 days post-pleural injection of 2 x 10^5 Lewis lung carcinoma (LLC) cells. Note the NF-κB reporter signal (κB.eGFP) over pleural tumors (outlines). n = 10/group; P,

probability, unpaired Student's t-test. (**E, F**) Representative photographic/bioluminescent image overlays with pseudocolor scale (**E**) and results summary (**F**) of chest bioluminescence of *NGL* mice with metastatic malignant pleural effusions at 14 days post-pleural injection of 2 x 10^5 B16F10 skin melanoma, MC38 colon adenocarcinoma, or LLC cells before (undrained) and after (drained) pleural catheter insertion and fluid removal. n = 5/group; P, probability, repeated measures two-way ANOVA; ns and *, $P > 0.05$ and $P < 0.05$, respectively, for pre-post drainage comparison. Data in (**B,D,F**) are given as raw data (circles), median and quartiles (lines), and kernel density distributions (violin plots).

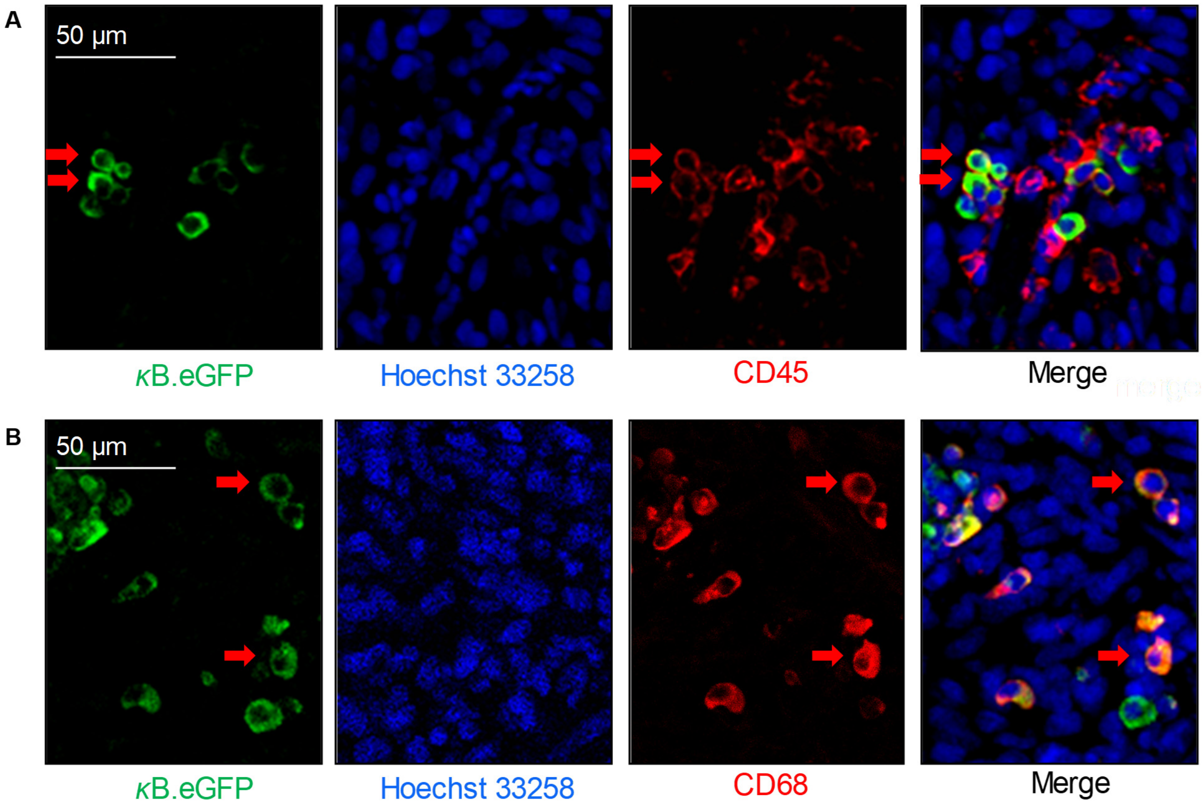

Figure S5. NF-κB activation in pleural metastases of *NGL* mice. Pleural metastases from mice treated as in Figure S4C,D show endogenous κB.eGFP reporter signal (green) that co-localizes with the pan-hematopoietic marker CD45 (**A**) and the macrophage marker CD68 (**B**) (both red fluorescent) in tumor-infiltrating myeloid cells and macrophages (arrows). Blue, nuclear Hoechst 33258 counterstaining.

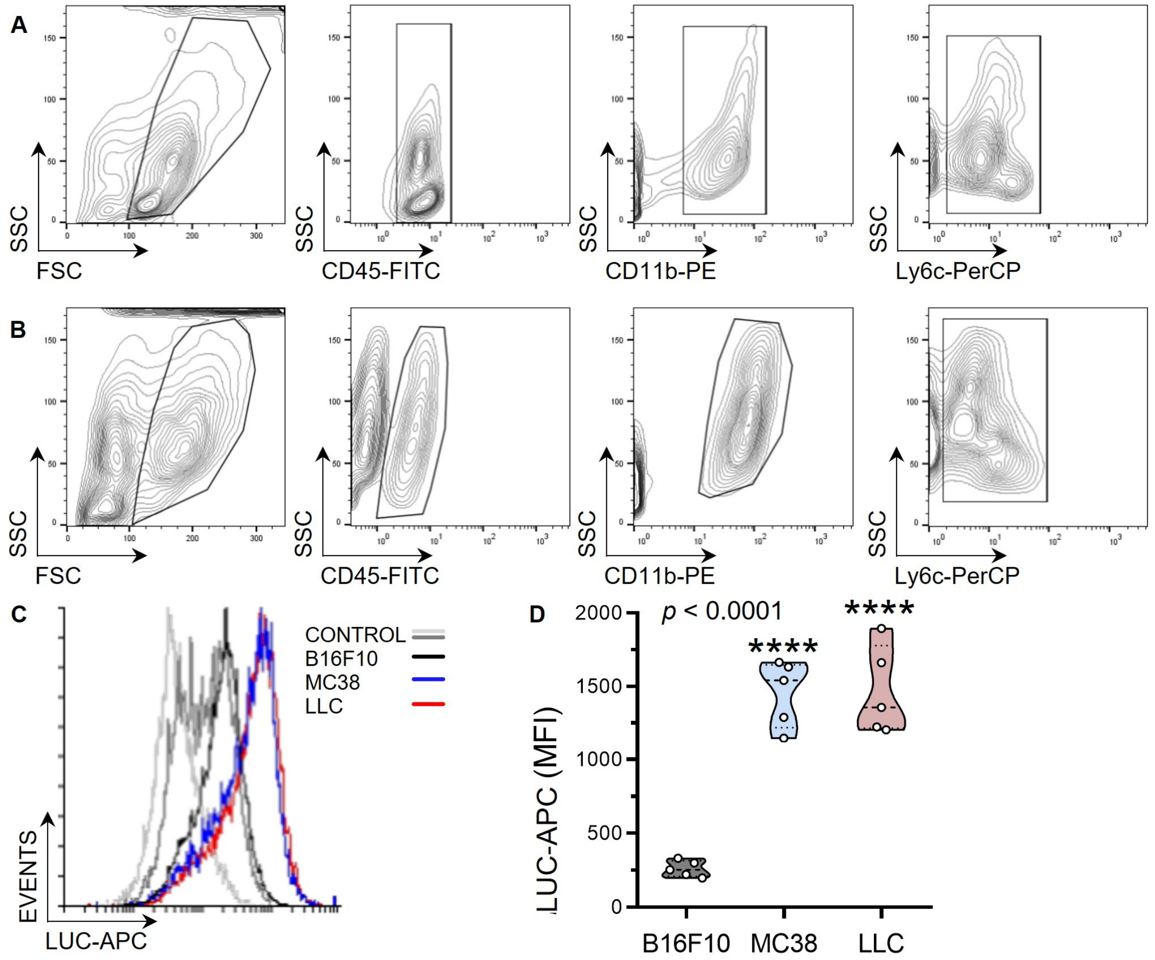

Figure S6. NF-κB activation in metastasis–associated macrophages. *NGL* NF-κB reporter mice received pleural PBS or DMEM (controls), or 2 x 10^5 B16F10 skin melanoma, MC38 colon adenocarcinoma, or Lewis lung carcinoma (LLC) cells and were sacrificed 14 days thereafter. Pleural fluid and pleural tumor cells were stained with antibodies against the hematopoietic marker CD45, myeloid markers CD11b and Ly6c, and the endogenous κB.LUC reporter. Representative flow cytometric dotplots showing the sequential gating strategy for macrophages of pleural fluid (**A**) and of tumors (**B**), and histogram (**C**) and data summary (**D**) of κB.LUC reporter signal in pleural macrophages. Data in (**D**) are given as raw data (circles), median and quartiles (lines), and kernel density distributions (violin plots). Sample size (n) = 5/group; P, probability, one-way ANOVA; ****, $P < 0.0001$ compared with mice injected with B16F10 cells, Bonferroni post-tests. MFI, mean fluorescence intensity.

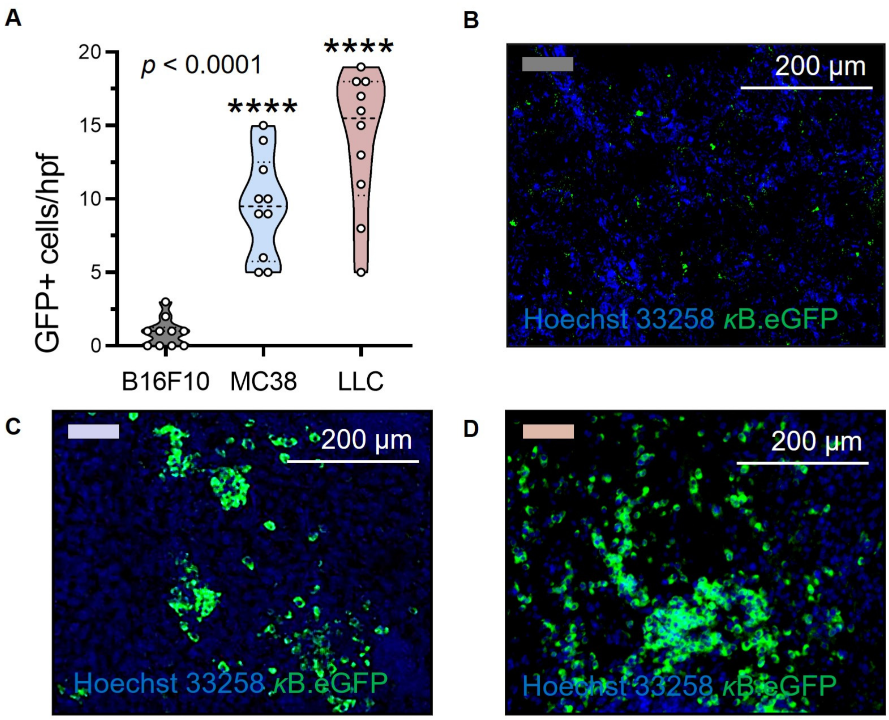

Figure S7. NF-κB activation in metastasis–associated macrophages. *NGL* NF-κB reporter mice received 2 x 10^5 pleural B16F10 skin melanoma, MC38 colon adenocar-cinoma, or Lewis lung carcinoma (LLC) cells and were sacrificed 14 days thereafter. Pleural tumors were assessed for κB.eGFP reporter signal by fluorescent microscopy after nuclear counterstaining with Hoechst 33258. Data summary (**A**) and representative merged microscopy images (**B-D**) of κB.eGFP+ pleural tumor cells. Data in (**A**) are given as raw data (circles), median and quartiles (lines), and kernel density distributions (violin plots). Sample size (n) = 10/group; P, probability, one-way ANOVA; ****, $P < 0.0001$ compared with mice injected with B16F10 cells, Bonferroni post-tests. hpf, high power field.

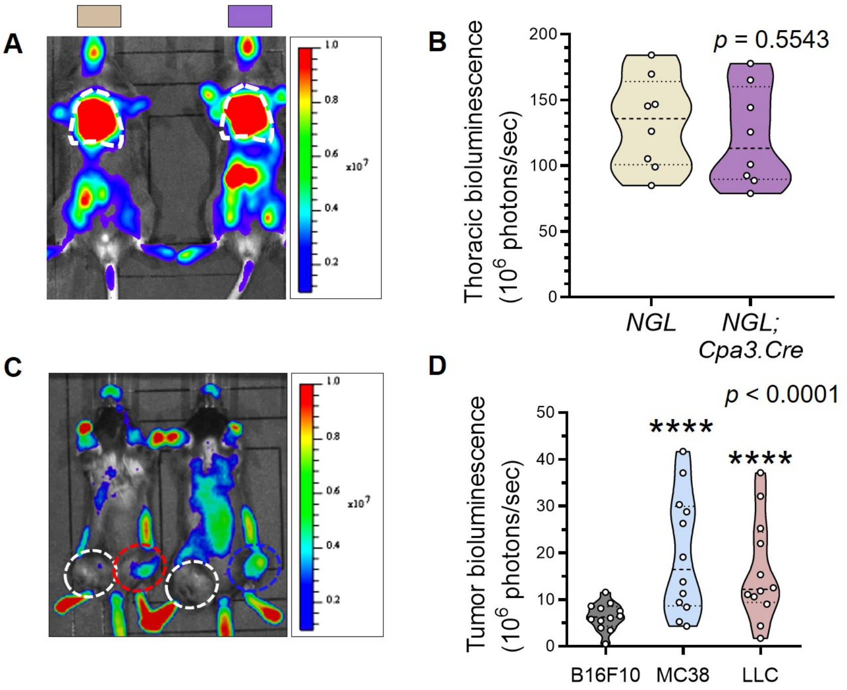

Figure S8. No impact of mast cells on the host NF-κB response to pleural metastasis and decreased intensity of the host NF-κB response to heterotopic tumor growth. (**A-B**) Representative photographic/bioluminescent image overlays with pseudocolor scale (**A**) and summary of results (**B**) from *NGL* mice additionally carrying no (*NGL*) or one (*NGL;Cpa3.Cre*) Cpa3.Cre allele that renders them, respectively, mast cell-competent or -deficient. Images and data were obtained at 14 days post-pleural injection of 2 x 10^5 Lewis lung carcinoma (LLC) cells, 5 min post-retroorbital injection of 1 mg D-Luciferin. Sample size (*n*) = 8/group; *P*, probability, unpaired Student's t-test. (**C-D**) Representative photographic/bioluminescent image overlays with pseudocolor scale (**C**) and summary of results (**D**) from *NGL* mice at 21 days post-subcutaneous injection of 5 x 10^5 B16F10 skin melanoma (white circles), MC38 colon adenocarcinoma (blue circles), or LLC (red circles) cells, 5 min post-retroorbital injection of 1 mg D-Luciferin. *n* = 12/group; *P*, probability, one-way ANOVA; ****, *P* < 0.0001 compared with B16F10 cells, Bonferroni post-tests. Data in (B, D) are given as raw data (circles), median and quartiles (lines), and kernel density distributions (violin plots).

A

Cell line	Expression	*Kras*
RAW264.7	-	WT
MC38	sh*C*	G13R
MC38	sh*Kras*	-
LLC	sh*C*	G12C
LLC	sh*Kras*	-

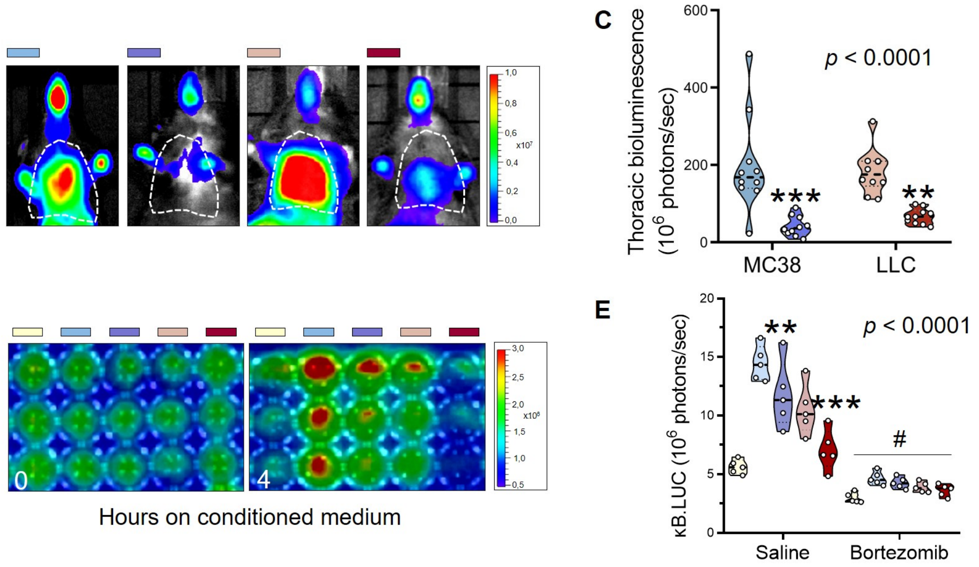

Figure S9. Requirement for mutant *Kras* signaling for host NF-κB activation during pleural metastasis. (**A**) Tumor cells with *Kras* mutations (MC38 and LLC cells) were engineered to stably express shRNA encoding random control (sh*C*) or anti-*Kras*-specific (sh*Kras*) sequences. *Kras*-wild-type RAW264.7 myelomonocytic leukaemia cells served as controls. Shown are color-coded tumor cell lines used with shRNA expression and *Kras* mutation status. (**B, C**) Tumor cells were injected into the pleural space of *NGL* mice (5 x 10^5/mouse). (**D, E**) RAW264.7 macrophages expressing p*NGL* plasmid were pretreated with saline or the proteasome and NF-κB inhibitor bortezomib (1 μg/mL equivalent to 3 μM), and were subsequently incubated with tumor-conditioned media (1:1 dilution in DMEM). Shown are representative photographic/bioluminescent images with pseudocolor scale (B, D) and results summaries of chest bioluminescence of *NGL* mice at 14 days post-tumor cells (C), and of cellular bioluminescence of p*NGL* RAW264.7 macrophages at 4 hours post-tumor-conditioned media (E). (C) n = 10/group; (E) n = 5 independent experiments/group; P, probability, two-way ANOVA; ** and ***, $P < 0.01$ and $P < 0.001$, respectively, compared with shC; #, $P < 0.05$ compared with the respective saline-pretreated cells. Data in (C, E) are given as raw data (circles), median and quartiles (lines), and kernel density distributions (violin plots).

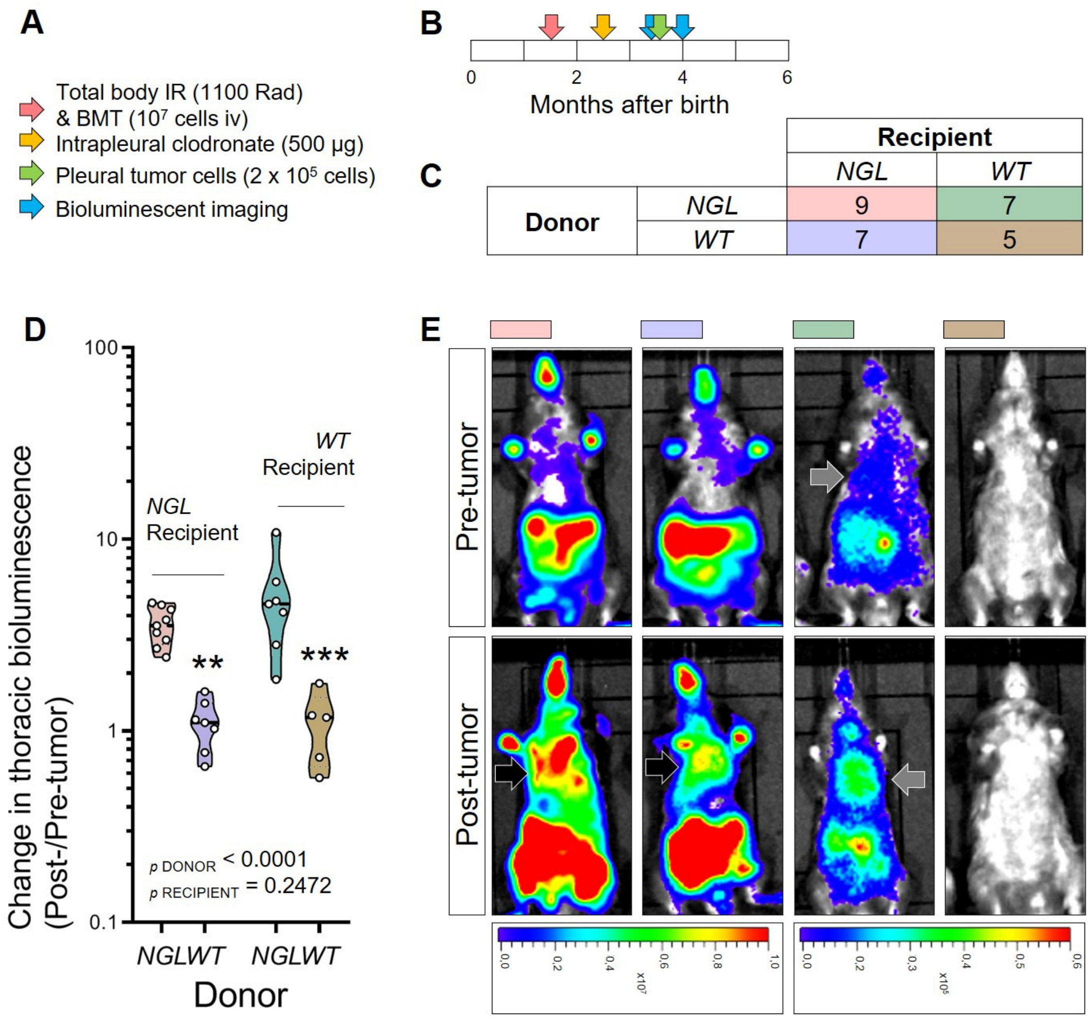

Figure S10. Adoptive bone marrow transplants determine host NF-κB response to pleural metastasis. *NF-κB.eGFP.LUC* reporter (*NGL*) and wild-type (*WT*) recipients received total–body irradiation (1100 Rad) followed by adoptive bone marrow replacement (BMT) from *WT* and *NGL* donors. After one month required for bone marrow chimerism, 500 µg liposomal clodronate was administered intrapleurally. After yet another month required for replacement of pleural myeloid cells by transplanted bone marrow cells, mice received 2 x 10^5 intrapleural LLC cells and were imaged for bioluminescence after 14 days. Shown are experimental schematic (each box represents one postnatal month) (**A, B**), color-coded table with experimental groups and sample size (*n*) (**C**), results summary (**D**), and representative photographic/bioluminescent images with pseudocolor scale taken at 5 min post-retroorbital injection of 1 mg D-Luciferin (**E**).

Data in (D) are given as raw data (circles), median and quartiles (lines), kernel density distributions (violin plots), and probability values (*P*), two-way ANOVA. ** and ***, $P < 0.01$ and $P < 0.001$, respectively, compared with mice that received BMT from *NGL* donors, Bonferroni post-tests.

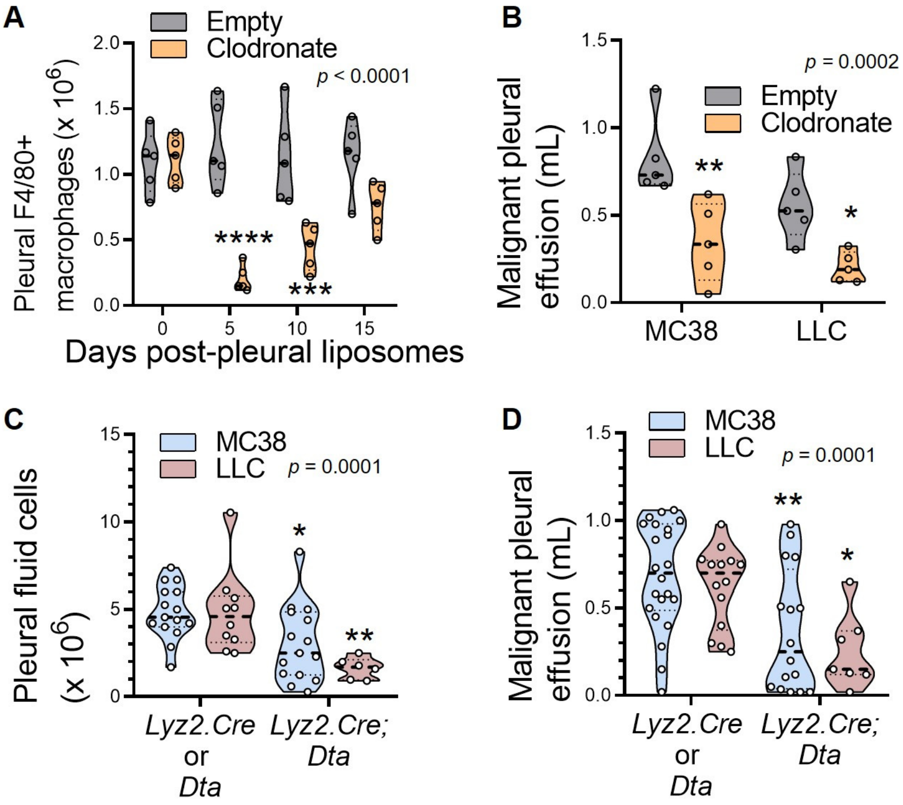

Figure S11. Pharmacologic and genetic macrophage ablation abolishes pleural metastasis. (**A**) *C57BL/6* mice (*n* = 5 mice/treatment/time-point) received intrapleural liposomal clodronate or empty liposomes (500 μg/mouse) and were subjected to pleural lavage at different time-points post-clodronate for analysis of macrophage numbers expressing F4/80 (encoded by *Adgre1*, the mouse orthologue of human *ADGRE1* from Fig. S1). *P*, probability, two-way ANOVA; *** and ****, $P < 0.001$ and $P < 0.0001$, respectively, compared with empty liposomes, Bonferroni post-test. (**B**) Pleural fluid volume of *C57BL/6* mice at 15 days post-intrapleural delivery of 500 μg liposomal clodronate or empty liposomes followed by 2 x 10^5 intrapleural MC38 or LLC cells one day thereafter and 500 μg liposomal clodronate or empty liposome re-administration into the pleural space 3 days later. *n* = 5 mice/group; *P*, probability, two-way ANOVA; * and **, $P < 0.05$ and $P < 0.01$, respectively, compared with empty liposomes, Bonferroni post-tests. (**C, D**) Pleural fluid nucleated cell number (**C**) and malignant pleural effusion volume (**D**) of *C57BL/6* mice carrying either one or both *Lyz2.Cre* and *Dta* alleles, at 14 days post-intrapleural delivery

of 2 x 10^5 MC38 or LLC cells. n = 22, 14, 16, and 7 mice/group from left to right; P, probability, two-way ANOVA; * and **, $P < 0.05$ and $P < 0.01$, respectively, for *Lyz2.Cre;Dta* macrophage-deficient mice compared with single transgenic controls, Bonferroni post-tests. All data are shown as raw data (circles), median and quartiles (lines), and kernel density distributions (violin plots).

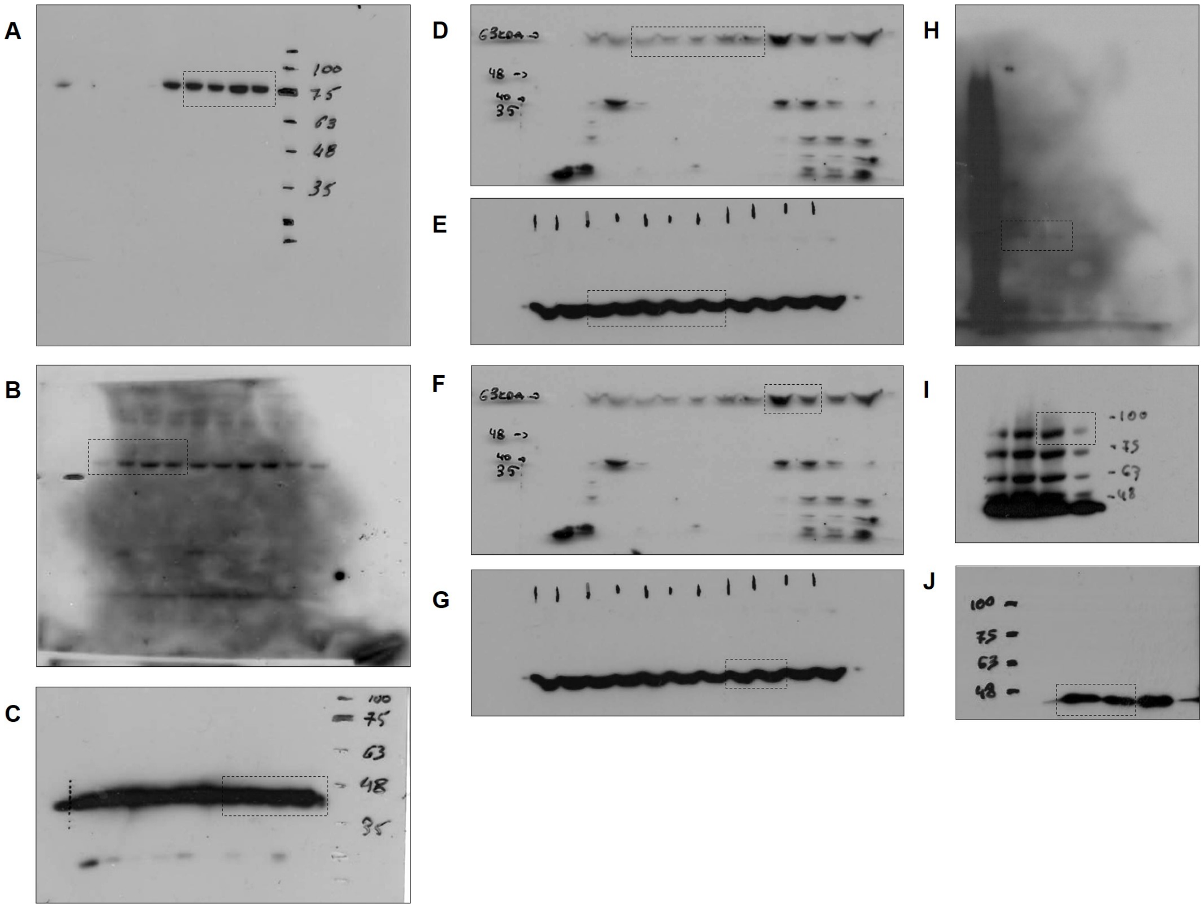

Figure S12. Uncropped immunoblots. Shown are uncropped immunoblots with areas displayed in main figures (dashed rectangles). (**A-C**) Blots shown in Figure 3C including IKKα (**A**), IKKβ (**B**), and β-actin (**C**). (**D, E**) Inverted blots shown in Figure 4E including versican (VCAN; **D**) and α-tubulin (TUBA; **E**). (**F-H**) Inverted blots shown in Figure 4H including IKKα (**H**), IKKβ (**F**), and β-actin (**G**). (**I, J**) Inverted blots shown in Figure 4I including VCAN (**I**) and TUBA (**J**).

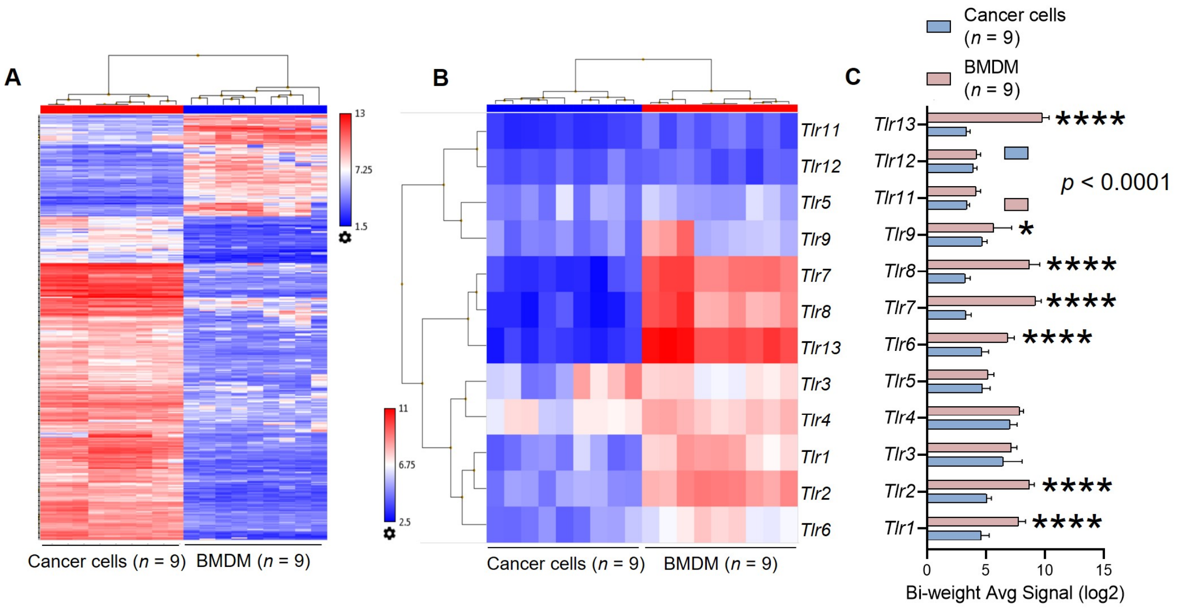
A
B
C
Cancer cells (n = 9)
BMDM (n = 9)
13
7.25
1.5
11
6.75
2.5
Tlr11
Tlr12
Tlr5
Tlr9
Tlr7
Tlr8
Tlr13
Tlr3
Tlr4
Tlr1
Tlr2
Tlr6
p < 0.0001
Tlr13 ****
Tlr12
Tlr11
Tlr9 *
Tlr8 ****
Tlr7 ****
Tlr6 ****
Tlr5
Tlr4
Tlr3
Tlr2 ****
Tlr1 ****
0
5
10
15
Bi-weight Avg Signal (log2)

Figure S13. Toll-like receptor (TLR) expression by murine bone marrow-derived macrophages (BMDM) by microarray. Murine BMDM from *C57BL/6* mice were isolated from whole bone marrow cells using one-week exposure to 20 ng/mL macrophage colony-stimulating factor (M-CSF). Total cellular RNA was extracted from BMDM under various conditions, as well as from different cancer cell lines (n = 9/group) and was hybridized to Affymetrix Mo Gene ST2.0 microarrays (data from GEO datasets GSE94847, GSE94880, GSE130624, and GSE130716). Shown are unsupervised clustering by all differentially expressed genes (**A**) and by TLR genes (**B**), as well as results summary for TLR gene expression (**C**). Data in (**C**) are shown as mean with SD; P, probability, two-way ANOVA; * and ****, $P < 0.05$ and $P < 0.0001$, respectively, compared with cancer cells, Bonferroni post-tests. Microarrays compared were GSM3744950, GSM3744952, GSM3744954, GSM3744955, GSM3744958, GSM3744961, GSM3744962, GSM3744957, and GSM3744960 (cancer cells) versus GSM2486425, GSM2486426, GSM2487750, GSM2487751, GSM3752396, GSM3752397, GSM3752398, GSM3752399, and GSM3752400 (BMDM).

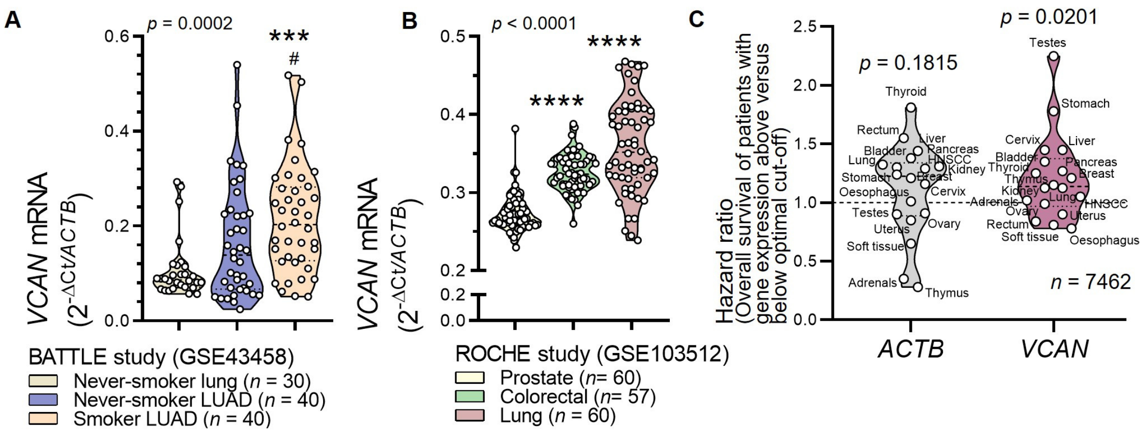

Figure S14. Versican as a potential diagnostic and prognostic biomarker of *KRAS*-mutant human cancers. (**A, B**) Data summary of *VCAN* expression normalized by *ACTB* transcripts in various human cancer types with different *KRAS* mutation frequencies ($KRAS^{MUT\%}$; data from COSMIC; https://cancer. sanger.ac.uk/ cosmic). (**A**) *VCAN/ACTB* expression in GEO dataset GSE43458 that encompasses lung adenocarcinomas (LUAD) from smokers ($KRAS^{MUT\%}$ = 36.5%) and never-smokers ($KRAS^{MUT\%}$ = 11.8%), as well as normal lung tissues from never-smokers ($KRAS^{MUT\%}$ < 1.0%). (**B**) *VCAN/ACTB* expression in GEO dataset GSE103512 that encompasses prostate ($KRAS^{MUT\%}$ = 2.8%), colorectal ($KRAS^{MUT\%}$ = 32.4%), and lung ($KRAS^{MUT\%}$ = 14.9%) cancers. (**A, B**) *n*, sample size; *P*, probability, one-way ANOVA; *** and ****, $P < 0.001$ and $P < 0.0001$, respectively, compared with lung tissue from never-smokers (**A**) or prostate cancer (**B**); #, $P < 0.05$ compared with LUAD from never-smokers, Bonferroni post-tests. **(C)** Kaplan-Meier survival analyses from all patients within the KMplot database stratified by *VCAN* transcript expression by optimal cut-offs (data from KMplot; http://kmplot.com/analysis/index.php?p=service&cancer=pancancer_rnaseq). Shown is summary of hazard ratios (HR) obtained for *VCAN* and *ACTB* control expression for 7,462 patients with 18 different tumor types. *P*, probability, one-sample Wilcoxon test for comparison with HR = 1.0. All data are shown as raw data (circles), median and quartiles (lines), and kernel density distributions (violin plots).

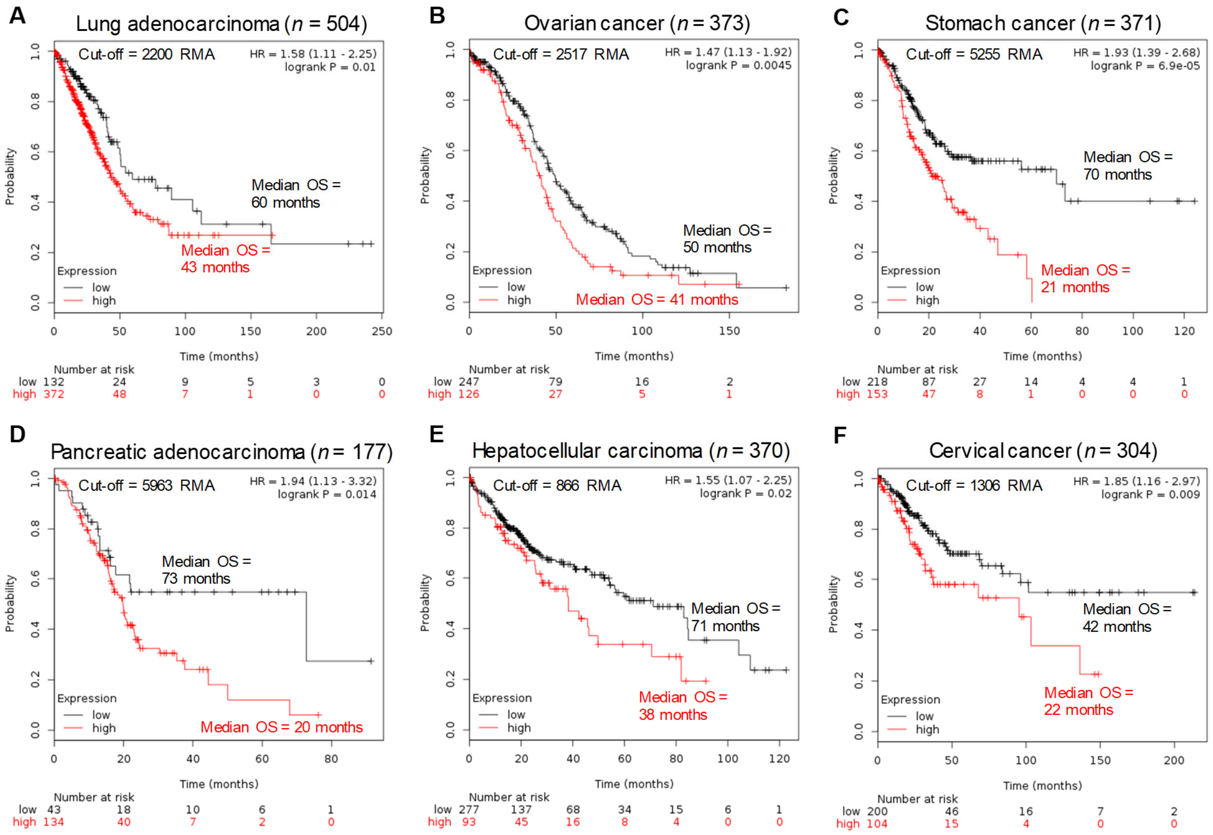

Figure S15. Versican over-expression by *KRAS*-mutant human cancers predicts poor survival. Kaplan-Meier survival plots with median overall survival (OS), hazard ratios (HR) with 95% confidence interval, and univariate log-rank probability values (*P*) from 504 patients with lung adenocarcinoma (**A**), 373 with ovarian cancer (**B**), 371 with stomach cancer (**C**), 177 with pancreatic cancer (**D**), 370 with liver cancer (**E**), and 304 with cervical cancer (**F**), stratified into low (black) and high (red) *VCAN* transcript expression by the optimal cut-offs indicated. RMA, robust microarray average (data from KMplot; http://kmplot.com/analysis/index.php?p=service&cancer=pancancer_rnaseq).

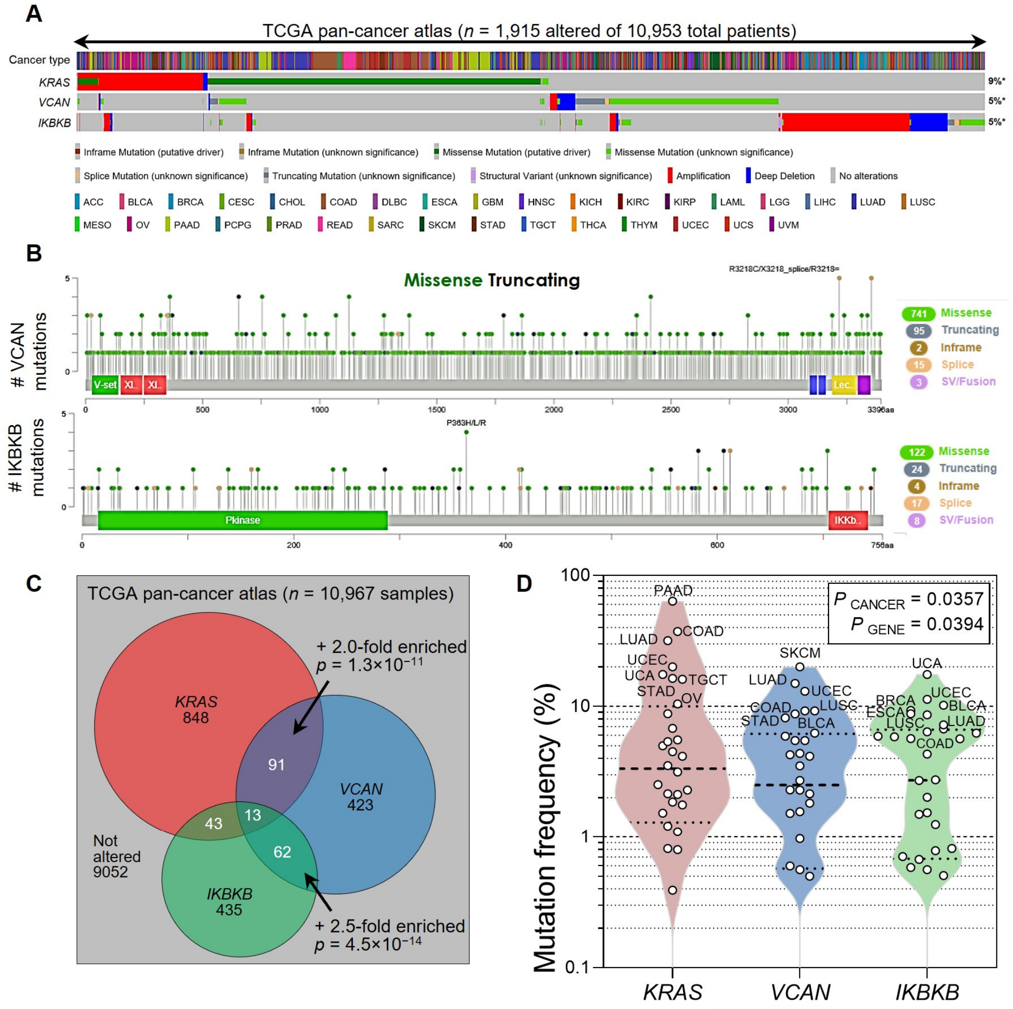

	VCAN		IKBKB	
n(%)	ρ	p	ρ	p
KRAS	0.5035	0.0033	0.4948	0.0040
VCAN	-	-	0.6479	6.1×10^{-5}

Figure S16. *KRAS*, *VCAN*, and *IKBKB* alterations in human cancers. *KRAS*, *VCAN*, and *IKBKB* mutation frequencies in the cancer genome atlas (TCGA) pan-cancer dataset (n = 10,967 samples from 10,953 patients). Data are from https://www.cbioportal.org/ (link: https://bit.ly/3yGys8i). Shown are mutation plot with alteration frequencies (**A**), lollipop plots (**B**), co-occurrence Venn diagram (**C**), and most frequently altered tumor types (**D**). In (**A**), columns represent patients and rows genes. In (**C**), shown are sample numbers (n). P, probability, hypergeometric test. In (**D**), shown are raw data points (circles), rotated kernel density distributions (violins), medians (dashed lines), quartiles (dotted lines), top quartile-altered cancer types with P, two-way ANOVA, and Spearman's correlation coefficients (ρ) and P across 32 cancer types. TCGA acronyms are from https://gdc.cancer.gov/resources-tcga-users/tcga-code-tables/tcga-study-abbreviations. Note the alteration enrichment (addiction) of *VCAN* to *KRAS* and of *IKBKB* to *VCAN* along the pathway proposed here. Note also that lung adenocarcinoma (LUAD), colon adenocarcinoma (COAD), and uterine corpus endometrial carcinoma (UCEC) are among the top 25% mutated cancers for all three genes and among the most frequent cancer types to metastasize to the pleural space.

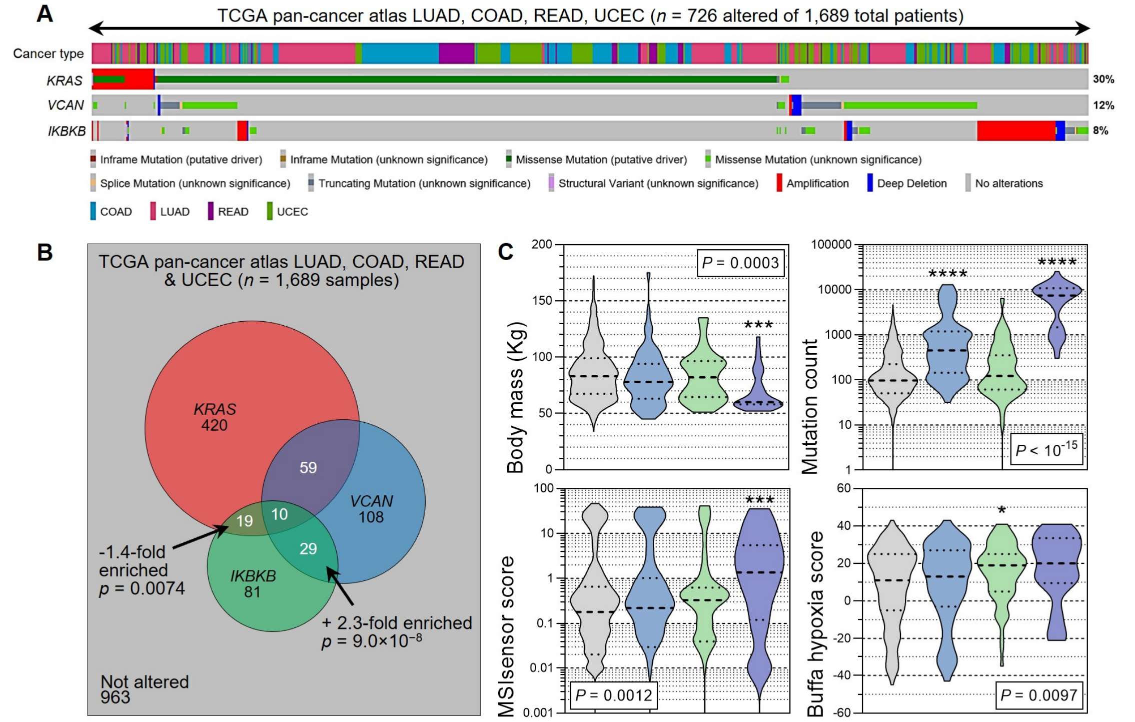

Enrichment of *VCAN/IKBKB* alterations in TCGA pan-cancer atlas LUAD, COAD, READ & UCEC (*KRAS* alteration rate = 30%) compared with total TCGA pan-cancer atlas dataset (*KRAS* alteration rate = 9%):
***VCAN*: + 2.27-fold ($p = 9\times10^{-34}$)**
***IKBKB*: + 1.63-fold ($p = 7\times10^{-10}$)**

n(%)	**Grade 1**	**Grade 2**	**Grade 3**	**High**	**p n(%)**
None	88(20)	101(23)	230(53)	8(1)	Ref
VCAN	6(14)	11(26)	25(59)	0(0)	0.5981(0.4674)
IKBKB	2(6)	3(9)	26(78)	2(6)	**0.0071(7×10^{-5})**
Both	1(3)	4(14)	21(77)	1(3)	0.0547**(0.0002)**

Overall p = 0.0144($p = 2.6\times10^{-6}$)

Figure S17. *VCAN* and *IKBKB* alterations in lung adenocarcinoma (LUAD), colon adenocarcinoma (COAD), rectal adenocarcinoma (READ), and uterine corpus endometrial carcinoma (UCEC). *KRAS*, *VCAN*, and *IKBKB* mutation frequencies in the cancer genome atlas (TCGA) LUAD, COAD, READ, and UCEC datasets (*n* = 1,689 samples/patients). Data are from https://www.cbioportal.org/ and can be retrieved at https://bit.ly/3wzrbFF. Shown are mutation plot with alteration frequencies (**A**), co-occurrence Venn diagram (**B**), and features of altered versus unaltered patients (**C**). In (**A**), columns represent patients and rows genes. In (**B**), shown are sample numbers (*n*). *P*, probability, hypergeometric test. In (**C**), shown are rotated kernel density distributions (violins), medians (dashed lines), quartiles (dotted lines), and UCEC patient numbers (*n*) and percentages (%). TCGA acronyms are from https://gdc.cancer.gov/resources-tcga-users/tcga-code-tables/tcga-study-abbreviations. *P*, probability, Kruskal-Wallis test (graphs) or overall and paired χ^2 tests for patient numbers and percentages before and in parentheses (table). *, ***, and ****: P < 0.05, P < 0.001, and P < 0.0001 compared with none-altered patients, Dunn's post-tests. In (**B**), note the alteration enrichment (oncogene addiction) of *IKBKB* to *VCAN* and the oncogene repulsion of *IKBKB* to *KRAS*. Note also the significant enrichment of *VCAN* and *IKBKB* alterations in the selected highly *KRAS*-mutant tumor types. In (**C**), note that *VCAN*-, *IKBKB*-, and *VCAN/IKBKB*-altered patients display varying degrees of cachexia (not recorded for LUAD), hypermutation, microsatellite instability (MSI), hypoxia, and advanced tumor grade.

Table S1. Differential gene expression of BMDM-specific transcripts after incubation with tumour-conditioned media. Transcripts statistically significantly (overall ANOVA *P* < 0.05) over-represented > 5-fold in bone marrow-derived macrophages (BMDM) compared with MC38 and LLC cancer cells and induced > 5-fold in BMDM after incubation with MC38 and LLC cell-conditioned media (CM), as assessed by microarray (mouse Gene ST2.0, Affymetrix, Sta.Clara, CA). Note the significant induction of *Il1b* shaded grey. Microarrays compared were GSM3744950, GSM3744952, GSM3744954, GSM3744955, and GSM3744958 (cancer cells) versus GSM2486425, GSM2487750, GSM3744964, GSM3752400, and GSM2486426 (naïve BMDM) versus GSM3752396, GSM3752397, GSM3752398, GSM3752399, and GSM2487751 (tumor-conditioned BMDM).

**Gene symbol**	**Gene name**	**ΔGE**[a]	**ΔGE**[b]	**ANOVA *p***[c]
Il1b	interleukin 1 beta	7.83	69.57	0.02770
Mmp8	matrix metallopeptidase 8	7.43	38.33	0.00002
Irg1	immunoresponsive gene 1	7.09	25.92	0.01569
Pf4	platelet factor 4	7.77	23.76	0.00101
Bst1	bone marrow stromal cell antigen 1	5.21	21.62	0.00018
Ccl12	chemokine (C-C motif) ligand 12	5.46	21.04	0.03587
Clec4n	C-type lectin domain family 4, member n	13.05	14.19	0.02143
Pilra	paired immunoglobin-like type 2 receptor alpha	7.92	12.89	0.00887

Ccl6	chemokine (C-C motif) ligand 6	50.15	8.39	0.00036
Gpr84	G protein-coupled receptor 84	13.52	7.39	0.02461
Pilrb1	paired immunoglobin-like type 2 receptor beta 1	5.99	5.76	0.00305
Wfdc17	WAP four-disulfide core domain 17	29.07	5.65	0.02023
Srgn	serglycin	20.98	5.10	0.00121

[a] ΔGE: average differential gene expression in unstimulated BMDM over cancer cells.
[b] ΔGE: average differential gene expression in cancer cell CM-incubated over unstimulated BMDM.
[c] ANOVA *p*: probability, one-way ANOVA.

Table S2. Differential gene expression of *Kras*-mutant cancer cells. Transcripts over-represented in MC38, LLC, and AE17 cells > 10-fold compared with PANO2 and B16F10 cells, as assessed by microarray (mouse Gene ST2.0, Affymetrix, Sta.Clara, CA). Only *Nid1* and *Vcan* (shaded grey) were also identified by the proteomic screen of tumour cell supernatants. Microarrays compared were GSM3744958 and GSM3752395 (*Kras*-wild-type cancer cells) versus GSM3744954, GSM3744950, and GSM3744952 (*Kras*-mutant cancer cells).

Gene symbol	Gene name	ΔGE[a]	ANOVA *p*[b]
Ccl2	chemokine (C-C motif) ligand 2	51.05	0.003345
Prrx1	paired related homeobox 1	49.20	0.000070
Pxdn	peroxidasin homolog (Drosophila)	48.21	0.000247
Gpr149	G protein-coupled receptor 149	45.96	0.004762
Hist1h1b	histone cluster 1, H1b	39.05	0.029208
Ogn	osteoglycin	38.41	0.009883
Hspb8	heat shock protein 8	27.71	0.002543
Ptprn	protein tyrosine phosphatase, receptor type, N	26.87	0.007407
Dkk2	dickkopf homolog 2 (Xenopus laevis)	25.44	0.007188
Apbb1ip	amyloid beta (A4) precursor protein-binding, family	24.82	0.007777
Epb41l3	erythrocyte membrane protein band 4.1 like 3	21.67	0.009291
Fbn1	fibrillin 1	19.89	0.022138
Ccl7	chemokine (C-C motif) ligand 7	19.20	0.003594
Col3a1	collagen, type III, alpha 1	18.25	0.046921
Flrt2	fibronectin leucine rich transmembrane protein 2	17.40	0.004230
Dhrs9	dehydrogenase/reductase (SDR family) member 9	16.82	0.011891
Nid1	nidogen 1	16.77	0.031483
Vcan	versican	14.94	0.012513
Dusp9	dual specificity phosphatase 9	13.97	0.000431
Syne1	spectrin repeat containing, nuclear envelope 1	12.97	0.009084
Chst11	carbohydrate sulfotransferase 11	12.35	0.013923
Fkbp10	FK506 binding protein 10	12.21	0.013300
Vcam1	vascular cell adhesion molecule 1	12.13	0.047450
Pdgfra	platelet derived growth factor receptor, alpha	10.88	0.032029
Clec1a	C-type lectin domain family 1, member a	10.09	0.005469

[a] ΔGE: average differential gene expression in MC38, LLC, and AE17 cells over PANO2 and B16F10 cells.
[b] ANOVA *p*: probability, one-way ANOVA.

Table S3. Differential gene expression of BMDMs lacking NF-κB signalling. Transcripts under-represented > 5-fold in bone marrow-derived macrophages (BMDM) from *Lyz2.Cre;Chukf/f* and *Lyz2.Cre;Ikbkbf/f* mice compared with *Lyz2.Cre* controls, as assessed by microarray (mouse Gene ST2.0, Affymetrix, Sta.Clara, CA). Note the significant down-regulation of *Lyz2* and *Il1b*. Microarrays compared were GSM2486425 (pooled BMDM from *Chukf/f*, *Ikbkbf/f*, and *Lyz2.Cre* mice) versus GSM3752396 (BMDM from *Chukf/f;Lyz2.Cre* mice) versus GSM3752397 (BMDM from *Ikbkbf/f;Lyz2.Cre* mice).

Gene symbol	Gene name	ΔGE[a]	ΔGE[b]	ΔGE[c]
Lyz2	lysozyme 2	-50.60	-67.00	-58.80
Uty	ubiquitously transcribed tetratricopeptide repeat gene, Y chromosome	-24.36	-25.20	-24.78
Hpgd	hydroxyprostaglandin dehydrogenase 15 (NAD)	-27.81	-9.50	-18.66
Kdm5d	lysine (K)-specific demethylase 5D	-12.95	-17.20	-15.08
Il1b	interleukin 1 beta	-8.78	-11.79	-10.29
Ifit1bl1	interferon induced protein with tetratricpeptide repeats 1B like 1	-14.28	-6.15	-10.22
Ddx3y	DEAD (Asp-Glu-Ala-Asp) box polypeptide 3, Y-linked	-11.14	-8.56	-9.85
C3	complement component 3	-10.08	-7.30	-8.69
Eif2s3y	eukaryotic translation initiation factor 2, subunit 3, structural gene Y-linked	-6.04	-7.91	-6.98
Ifi44	interferon-induced protein 44	-5.93	-7.88	-6.91
Tmem176a	transmembrane protein 176A	-6.78	-5.75	-6.27

[a] ΔGE: average differential gene expression in BMDM from *Lyz2.Cre;Chukf/f* mice over *Lyz2.Cre* controls.
[b] ΔGE: average differential gene expression in BMDM from *Lyz2.Cre;Ikbkbf/f* mice over *Lyz2.Cre* controls.
[c] ΔGE: average differential gene expression in BMDM from *Lyz2.Cre;Chukf/f* and *Lyz2.Cre;Ikbkbf/f* mice compared with *Lyz2.Cre* controls.

Table S4. PCR primers used in this study.

Assay[a]	Primer	Sequence	Product (bp)
PCR	*Kras* exon 1F	TCTTAGAGTTTTACACACAAAGGTGAG	289
PCR	*Kras* exon 1R	TTCAAAGCGGCTGGCTGC	
PCR	*Kras* exon 2F	TTGAGCTGTTTACATCACCTTG	315
PCR	*Kras* exon 2R	GCAAAGAATCAATAAATGTAAGCTATC	
PCR	*Kras* exon 3F	GTTGAGGGCAAGGTTTGAAG	287
PCR	*Kras* exon 3R	ACGAACACCATCTGGAGCAC	
PCR	*Mycoplasma Spp*. F	GGGAGCAAACAGGATTAGATACCCT	270
PCR	*Mycoplasma Spp*. R	TGCACCATCTGTCACTCTGTTAACCTC	
qPCR	m*Vcan* F	CCAGAACGGAAATATCAAGATTGG	201
qPCR	m*Vcan* R	TGAAACACAACACCATCCAC	
qPCR	m*Il1b* F	TTTGACAGTGATGAGAATGACC	162
qPCR	m*Il1b* R	AATGAGTGATACTGCCTGCC	
qPCR	m*Tnf* F	GTCCCCAAAGGGATGAGAAGT	124
qPCR	m*Tnf* R	TTTGCTACGACGTGGGCTAC	
qPCR	m*Gusb* F	TTACTTTAAGACGCTGATCACC	165
qPCR	m*Gusb* R	ACCTCCAAATGCCCATAGTC	
qPCR	h*ACTB* F	GAGCACAGAGCCTCGCCTTT	70
qPCR	h*ACTB* R	TCATCATCCATGGTGAGCTGG	
qPCR	h*VCAN* F	GAATGTCACTCTAATCCCTGTC	117
qPCR	h*VCAN* R	TGTCTCGGTATCTTGCTCAC	

[a]Assay: PCR, DNA polymerase chain reaction; qPCR, quantitative (real-time) PCR.
Provider: VBC Biotech, Vienna, Austria.

Table S5. Lentiviral shRNA pools used in this study.

Target	Abbreviation	Catalog #	Target Sequences
random	sh*C*	sc-108080-V	target sequence proprietary
Vcan	sh*Vcan*	sc-41904–V	5’-CAACCACCATCGAAATGAA-3’<br>5’-CTCACAGGCTGTAATTACA-3’<br>5’-GTAGACATATCCCATACTA-3’
Kras	sh*Kras*	sc-43876-V	5’-CTACAGGAAACAAGTAGTA-3’<br>5’-GAACAGTAGACACGAAACA-3’<br>5’-CCATTCAGTTTCCATGTTA-3’
Chuk	sh*Chuk*	sc-29366-V	5’-CCATGGTGTTTGAATGTATTT-3’<br>5’-CTCTCAGTGTGTTCTAGATTT-3’<br>5’-GCAAGCAGAAGATTATTGATT-3’
Ikbkb	sh*Ikbkb*	sc-35645-V	5’-GATGACATCTTGAACTTGATT-3’<br>5’-CTGCACATTTGAATCTGTATT-3’<br>5’-CAGCTCTCTTAGACAGTTATT-3’
Ikbke	sh*Ikbke*	sc-39057-V	5’-GAGATCATGTACAGAATCATT-3’<br>5’-CAGTGTTGTTTGGACAAGATT-3’<br>5’-CCAACAAACTAGCATTACTTT-3
Tbk1	sh*Tbk1*	sc-39059-V	5’-GTAGGACTGAGATATGAAATT-3’<br>5’-GCATCACAGAGATTTACTATT-3’<br>5’-GAAGTTCTAGTTTGCACAATT-3’

Provider: Santa Cruz Biotechnology, Dallas, TX.

Data S1.

*.xlsx file of proteomic analysis of secreted proteins of *Kras*-mutant and -wild-type cancer cells.
